# Resolving the contribution of the uncoupled phycobilisomes to cyanobacterial pulse-amplitude modulated (PAM) fluorometry signals

**DOI:** 10.1007/s11120-015-0141-x

**Published:** 2015-04-19

**Authors:** Alonso M. Acuña, Joris J. Snellenburg, Michal Gwizdala, Diana Kirilovsky, Rienk van Grondelle, Ivo H. M. van Stokkum

**Affiliations:** Institute for Lasers, Life and Biophotonics, Faculty of Sciences, VU University Amsterdam, De Boelelaan 1081, 1081 HV Amsterdam, The Netherlands; Institut de Biologie et Technologies de Saclay (iBiTec-S), Commissariat à l’Energie Atomique et aux Energies Alternatives (CEA), 91191 Gif-sur-Yvette, France

**Keywords:** Pulse-amplitude modulated (PAM) fluorometry, Fluorescence quantum yield, Non-photochemical quenching, Phycobilisome, Cyanobacteria

## Abstract

Pulse-amplitude modulated (PAM) fluorometry is extensively used to characterize photosynthetic organisms on the slow time-scale (1–1000 s). The saturation pulse method allows determination of the quantum yields of maximal (*F*_M_) and minimal fluorescence (*F*_0_), parameters related to the activity of the photosynthetic apparatus. Also, when the sample undergoes a certain light treatment during the measurement, the fluorescence quantum yields of the unquenched and the quenched states can be determined. In the case of cyanobacteria, however, the recorded fluorescence does not exclusively stem from the chlorophyll *a* in photosystem II (PSII). The phycobilins, the pigments of the cyanobacterial light-harvesting complexes, the phycobilisomes (PB), also contribute to the PAM signal, and therefore, *F*_0_ and *F*_M_ are no longer related to PSII only. We present a functional model that takes into account the presence of several fluorescent species whose concentrations can be resolved provided their fluorescence quantum yields are known. Data analysis of PAM measurements on in vivo cells of our model organism *Synechocystis* PCC6803 is discussed. Three different components are found necessary to fit the data: uncoupled PB (PB_free_), PB–PSII complexes, and free PSI. The free PSII contribution was negligible. The PB_free_ contribution substantially increased in the mutants that lack the core terminal emitter subunits allophycocyanin D or allophycocyanin F. A positive correlation was found between the amount of PB_free_ and the rate constants describing the binding of the activated orange carotenoid protein to PB, responsible for non-photochemical quenching.

**Electronic supplementary material:**

The online version of this article (doi:10.1007/s11120-015-0141-x) contains supplementary material, which is available to authorized users.

## Introduction

**Fig. 1:**
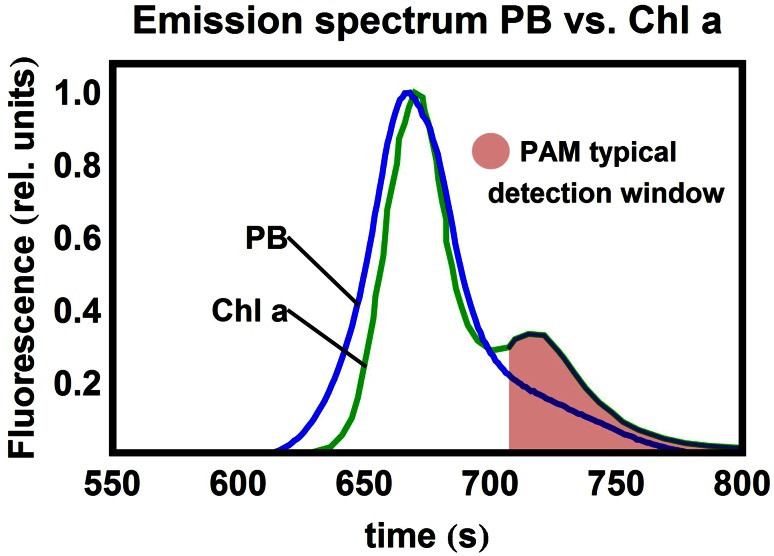
Emissive spectra of Chl *a* and PB in vivo. The spectral overlap within the detection region of typical PAM setups is shown in *red*

Chlorophyll *a* (Chl *a*) fluorescence carries important information about the primary photophysical processes taking place in the thylakoid membrane, including non-photochemical quenching (NPQ) (Baker 2008; Krause and Weis 1991; Papageorgiou and Govindjee 2004; van Grondelle 1985; van Grondelle et al. 1994). This is why Chl *a* fluorescence quenching analysis by the saturation pulse method has been extensively used in the study of photosynthetic organisms (Schreiber et al. 1995). Pulse-amplitude modulated (PAM) fluorometry is a sensitive tool which measures the fluorescence quantum yield *ϕ*_Fl_ in different sample states, *e.g.* quenched/unquenched. This has enabled in vivo characterization of the photosynthetic apparatus of *Synechocystis* PCC6803 (hereafter *Synechocystis*) in different light-acclimated states (Campbell et al. 1998; Kirilovsky 2007, 2014; Wilson et al. 2007) In the particular case of cyanobacteria, the recorded fluorescence originates from different sources: not only Chl *a* of photosystems (PSI and PSII) but also phycobilins, embedded in the antenna complexes, the phycobilisomes (PBs), contribute to the PAM signal (see Fig. 1 and reviews Campbell et al. 1998; Kirilovsky 2014.

Therefore, a mathematical model is needed in order to interpret the data correctly. Holzwarth et al. have already emphasized that PAM fluorescence data often get converted to the (Stern–Volmer) NPQ space via a simple, but by no means “innocent”, mathematical transformation x(*t*) → x^−1^(*t*) leading to distorted kinetics (Holzwarth et al. 2013). Instead, a careful description of the hypothesized NPQ processes is essential. An example of such PAM modelling efforts is the kinetic model developed by Ebenhöh and co-workers for zeaxanthin-related NPQ in plants and from which PAM curves could be simulated (Ebenhöh et al. 2011). However, systematic discrepancies between their simulations and experimental PAM data, in particular in the light to dark transition, have shown just how challenging it is to consistently reproduce the NPQ kinetics while validating strongly simplifying assumptions. In another attempt, the rapidly reversible component of NPQ and its dynamically controlled appearance/disappearance under low and strong actinic light conditions have been modelled by Zaks et al. (2012). Finally, models have been developed that combine detailed PSII and inter-photosystem electron transport with Calvin-cycle reactions (Laisk et al. 1997). In this article, we focus on cyanobacterial PAM studies in which the PB-related contribution to the PAM signal must first be resolved for, as long as it remains neglected, no solid statements on the PSII-specific activity can be formulated.

### Phycobilisomes: the difference between cyanobacterial and plant signals

**Fig. 2:**
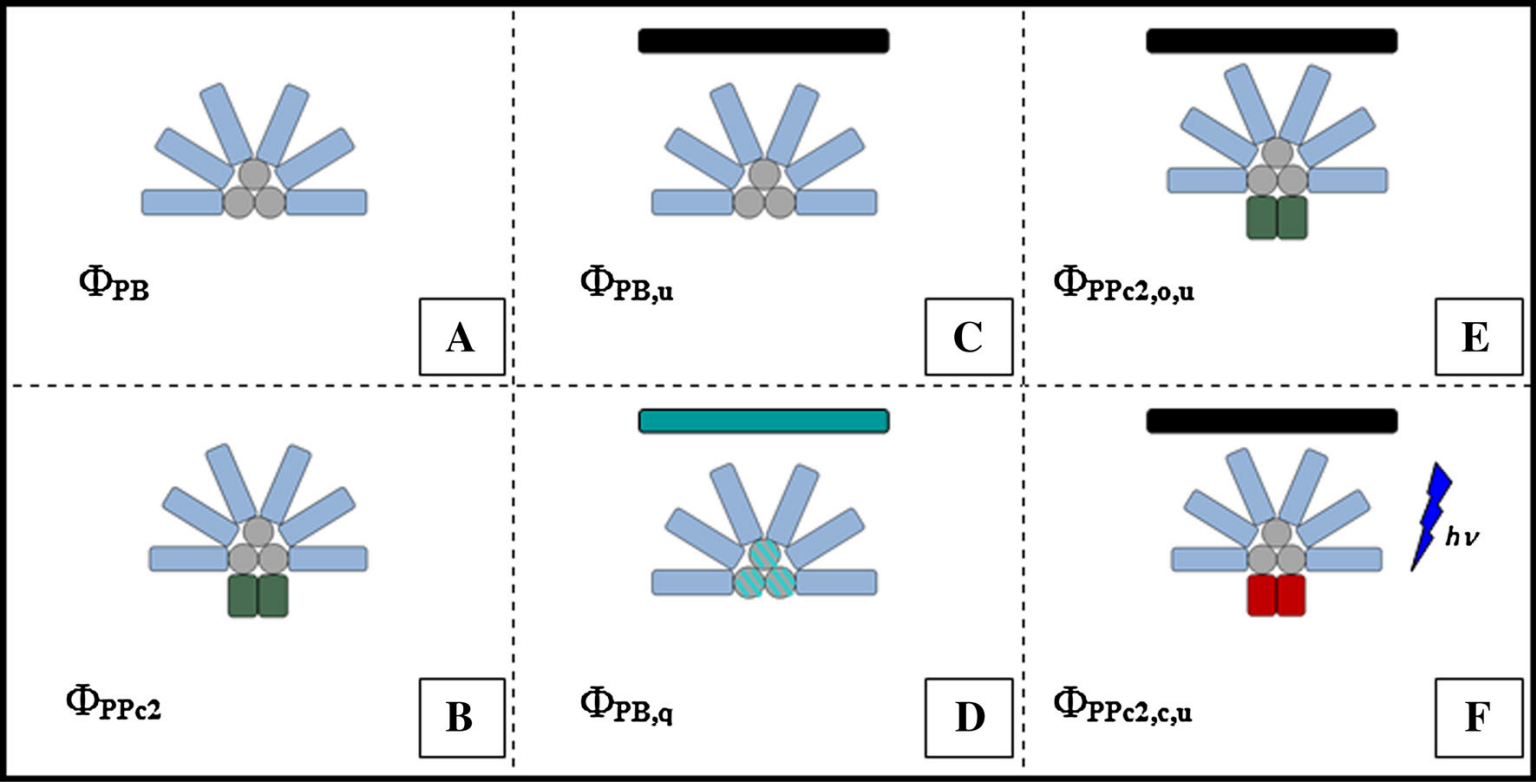
The different emissive species giving rise to a cyanobacterial PAM signal [see Eq. (3)] are characterized by their respective quantum yields in different light-acclimated states. Phycobilisomes are represented as *six blue rods* attached to *three grey cylinders* as the core. Two rectangular units represent an open (*dark green*) or closed (*red*) PSII-dimer. PBs can be bound to a PS or not. The index *j* indicates whether the emissive species is an uncoupled PB (**a** *j* = PB) a single PB–PSII complex (PPc2) (**b** *j* = PPc2). *j* = PB can still result in two different states if, for instance, a minimal amount of light (*black bar*) or high-light (*blue–green bar*) is shone onto the sample: unquenched (**c** *l* = u) or quenched (**d** *l* = q). The index *k* indicates whether the PSII is open (**e** *k* = o) or, in case a saturation pulse fully reduces the PQ pool, closed (**f** *k* = c)

The phycobilisome is the cyanobacterial light-harvesting complex. In *Synechocystis*, the phycobilisomes are composed of six C-phycocyanin (C-PC) rods (see Fig. 2) each of which is composed on average of three disc-shaped hexamers (12 bilins per hexamer) (Arteni et al. 2009) that absorb at ca. 620 nm and whose fluorescence maxima lie within the range 640–650 nm (Glazer et al. 1983). These C-PC rods are linked to the three allophycocyanin (APC) core cylinders arranged such that their respective centres draw a triangle with one of its sides facing the thylakoid membrane (Bryant et al. 1979). In each of the core cylinders, there are four trimer discs (six bilins per trimer). Each monomer of the trimer is made of two polypeptidic units called *α*^APC^ and *β*^APC^; their absorption peak lies at 650 nm and they emit at 660 nm, hence why they are referred to as APC_660_. However, within the basal cylinders, and in the proximity of the membrane, there are (i) one trimer in which one of the three monomers is not formed by one *β*^APC^ and one *α*^APC^-unit but one *β*^APC^ and one ApcD-unit instead (previously called *α*^APC-B^), (ii) a second trimer that also differs from the (*αβ*)_3_ conformation, where the *β*^APC^ is replaced by an ApcF (*β*^APC-*β*18^) and the *α*^APC^ by an ApcE (the core-membrane linker, *α*^Lcm^) (see reviews by Adir 2005; MacColl 1998). Even though the linker proteins do not contain pigments themselves, they have been shown to play a role in fine-tuning rod energy absorption and energy transfer capabilities (David et al. 2011)

### NPQ in Synechocystis

PB-containing cyanobacteria (such as *Synechocystis*) have developed different non-photochemical-quenching photoprotective mechanisms. The two principal ones are state transitions and OCP-related NPQ. The former denotes the organism’s ability to distribute excitation energy between PSI and PSII as a function of the redox status of the linear electron transport chain (Fork and Satoh 1986; Kirilovsky et al. 2014; Mullineaux and Allen 1990; Vernotte et al. 1990). When cyanobacteria cells are illuminated with orange light, principally absorbed by PBs, the PQ pool becomes more reduced and a transition to state 2 is induced accompanied by a decrease of fluorescence. In contrast, blue light, principally absorbed by chlorophyll, induces a transition to state 1 and increase of fluorescence. In darkness, *Synechocystis* are known to be in state 2, since the PQ pool is reduced via the respiration. Thus, illumination of dark-adapted cells with low intensities of blue light provokes a transition to state 1. Modelling state transitions, however, goes beyond the scope of this study. Instead, we will focus on the second mechanism, the OCP-related NPQ. In higher plants, the energy-related component of NPQ is induced by a pH-gradient (Wraight and Crofts 1970) and involves the xanthophyll cycle (Demmig et al. 1987) and/or the PsbS protein protonation (Funk et al. 1995; Li et al. 2000). In contrast, in cyanobacteria, an equivalent mechanism which reduces the energy arriving to the reaction centres is triggered when the orange carotenoid protein (OCP) is photoactivated and binds to the phycobilisomes (Gwizdala et al. 2011; Wilson et al. 2006, 2008). Binding only occurs if the non-active orange form OCP^o^ is photo-converted to the red form OCP^r^ (OCP^o^ → OCP^r^ conversion) (Gwizdala et al. 2011; Wilson et al. 2008). Thus, when strong blue–green light is absorbed by the carotenoid, OCP^r^ is first formed, and then it attaches to the PB core leading to most of the excitation energy being dissipated and not reaching the RCs. Since energy dissipation occurs at the PB level, we do not model NPQ at the dark and at the maximum fluorescence level differently, as it has been proposed for plants (Härtel and Lokstein 1995). Detaching of OCP is assisted by yet another protein: the fluorescence recovery protein (FRP) (Boulay et al. 2010; Sutter et al. 2013). The OCP/FRP ratio in the cell can be considered as the regulator of the induction of heat dissipation (Gwizdala et al. 2013).

### PAM fluorometry

Both photochemistry yields and NPQ induction can be followed by PAM fluorometry. A PAM instrument is able to trace changes of the yield of fluorescence under different light conditions. Typically, three levels of fluorescence, called *F*_0_, *F*_M_ and *F*_S_, are observed in a PAM trace. The minimal fluorescence level (*F*_0_) is recorded, while the photosynthetic organism is dark-adapted and all the PSII centres are open (the primary quinone *Q*_A_ is oxidized). The intensity of the modulated measuring light (in PAM 101–103: *λ*_m_ = 650 nm; dual-PAM: *λ*_m_ = 630 and 440–460 nm) used to determine *F*_0_ is very low (non-actinic) in order to avoid photochemistry, which could yield a biased minimum. The *F*_M_ level is induced by a saturation pulse typically of >2000 μmol of photons m^−2^ s^−1^. Its duration (200–1000 ms) ensures multiple turnovers and a transient closure of the reaction centres (*Q*_A_ reduced). Therefore, *F*_M_ corresponds to the state of fully closed PSII reaction centres (*Q*_A_ reduced). During this state, the yield of photochemistry is minimal, i.e. the recorded fluorescence level is maximal (Papageorgiou et al. 2007). The steady-state fluorescence level (*F*_S_) is measured under continuous illumination and depends on the fraction of reduced *Q*_A_. Other parameters, such as the variable fluorescence, *F*_V_, are derived from the measured values. *F*_V_ is defined as the difference between the maximal fluorescence level, *F*_M_, and the darkness fluorescence level (Butler 1978), *F*_0_: *F*_V_ = *F*_M_–*F*_0_ (see Fig. S1).

The phycobilins of the PBs, not present in higher plants, lead to a major difference in the PAM signal: since the Chl *a* (PSII) and the phycobilin (PB) fluorescence spectra overlap (see Fig. 1) precisely within the spectral region where fluorescence is typically collected (*λ*_det_ > 700 nm), *F*_0,cyano_ lies higher than the *F*_0_-level recorded in plants:

1 $$F_{{0,{\text{cyano}}}} = F_{{0,{\text{PSII}}}} + F_{{0,{\text{PB}}}} ,$$

where $F_{{0,{\text{PSII}}}} ,F_{{0,{\text{PB}}}}$ are, respectively, the fluorescence from PSII Chl *a* and from PBs. *F*_0,PB_ also adds to the maximal fluorescence:

2 $$F_{\text{M,cyano}} = F_{\text{M,PSII}} + F_{{ 0 , {\text{PB}}}} .$$

The ratio Chl *a*/phycobilin fluorescence depends on the wavelength *λ*_m_ of the measuring light. The amount of Chl *a*-related fluorescence when induced by blue light (exciting more the Chl *a* than the bilins) is greater than it is when orange light (specific to phycocyanin) is used instead. All measurements shown in this article were carried out using a PAM 101–103 instrument with red measuring light (*λ*_m_ = 650 nm) which yields an intermediate situation (Campbell et al. 1998; Kirilovsky 2014).

## A model to resolve several contributions to a cyanobacterial PAM signal

We propose a model that determines the *F*_0,PB_-related contribution to the PAM signal. In this way, one could resolve the PAM-dynamics strictly related to PSII. Our model is based on the fundamental assumption that the fluorescent species’ spectral properties are independent from its concentration in the sample. Any given PAM signal J_PAM_ can then be written as the product of an emissive species *j*, (spectrally) characterized by its fluorescence quantum yield *Φ*_*j*_, multiplied by its concentration *c*_*j*_ (which may or may not be time-dependent). Additionally, fluorescent species may be unquenched (dim blue light regime) or quenched (strong blue light regime).

A cyanobacterial sample will expectedly contain more than only one fluorescent species, so the PAM signal then becomes a linear superposition of the fluorescence originating from each one of the species. Furthermore, *J*_PAM_ depends on the light intensity of the measuring light, *I*(*t*), and a set of model parameters **P** = (*P*_1_,*P*_2_,*P*_3_…) which we will define further below (see “Time evolution” section). Thus, the general expression is

3 $$J_{\text{PAM}} (I(t);{\mathbf{P}}) = \sum\limits_{jkl} {c_{jkl} (t) \times \varPhi_{jkl} } ,$$

where the additional indices *k* and *l* represent different light-acclimated states; *k* takes into account whether PSII is open or closed and *l* stands for either a quenched or an unquenched species (see Fig. 2).

As a first example, consider two different species: let the first one be a phycobilisome–Photosystem II complex, abbreviated PPc2, while the second one, denoted PB_free_, represents either completely free PB or PB non-functionally bound to membranes and finally PB that do not transfer energy efficiently to the PS and therefore have a long fluorescence lifetime. Note that the PPc2 contribution contains both emission from Chl *a* and from the PB attached to PSII.

Defining the time-independent, relative concentrations as *c*_2_ (PPc2) and *γ* (PB_free_) and using Eq. (3), it follows for *F*_0,cyano_ and *F*_M,cyano_:

4 $$F_{{0,{\text{cyano}}}} = c_{2} \times \phi_{{{\text{PPc}}2,o ,u}} + \gamma \times \phi_{{{\text{PB,}}u}} ,$$

5 $$F_{\text{M,cyano}} = c_{2} \times \phi_{{{\text{PPc}}2,c ,u}} + \gamma \times \phi_{{{\text{PB,}}u}} .$$

Therefore, a model that aims to estimate the two unknown concentrations *c*_2_ and *γ* needs a priori knowledge with respect to the quantum yields *ϕ*_PB,*u*_, *ϕ*_PPc2,*o*,*u*_ and *ϕ*_PPc2,*c*,*u*_. Provided the latter are known, Eqs. (4)–(5) simply build a system of two equations with two unknowns.

This observation brings us to one of the fundamental assumptions in this article: the constant values for the fluorescence quantum yields of each species displayed in Table 1. These are derived from fluorescence lifetimes and emission spectra obtained from target analysis of time-resolved experiments. From streak camera studies performed on whole cells of *Synechocystis* combined with global and target analysis, the fluorescence lifetimes and emission spectra of the species present in the cell have been estimated (Tian et al. 2011, 2012, 2013). Table 1 thus provides the set of constant values needed to solve the system, and estimate the unknown concentrations. The numbers in this table should be considered an educated guess based upon the literature. The sensitivity of the results to these numbers has been investigated as well.

**Table 1:** Normalized estimates of each of the emissive species’ fluorescence quantum yields as determined from time-resolved spectroscopy and global and target analysis

	Unquenched	Quenched
Free PB	1	0.105
Free PSII (closed)	0.519	–
Free PSII (open)	0.036	–
Free PSI	0.023	–
PPc2 (closed)	0.502	0.189
PPc2 (open)	0.161	0.100
PPc1	0.106	0.060

## Results and discussion

### Parameter estimation from simulated data

Using the model described above, we first simulate data assuming a certain amount of noise and a given saturation pulse frequency (*spf*). Since the range of values for both quantum yields and relative concentrations is [0,1], we introduce a scaling factor that matches the absolute scale of the fluorescence recorder used during the experiment. Figure S2 shows simulated data with a noise level of 0.01 and a pulse every 50 or 20 s. The parameters were set to *γ* = 0.10 and *scale* = 2000. This places the *F*_0_ level at (0.10 × **1** + 0.90 × **0.161**) × 2000 = 488 arbitrary units (bold numbers from Table 1).

Fitting the simulated data yielded the following parameters: (a) *spf* (50 s)^−1^: *γ* = (0.1003 ± 0.004); *scale* = (1998.2 ± 2.5) and (b) *spf* (20 s)^−1^: *γ* = (0.1002 ± 0.003); *scale* = (1999.0 ± 1.9). The relative errors of the estimated parameters improved after increasing the *spf* (due to better sampling of the closed state). Hence, in an experiment where *F*_M_ should be determined precisely, one could increase the frequency at which the sample gets saturated. However, an *F*_M_ level decrease has been reported due to NPQ induced by the saturation pulses; this has been indeed observed in *Chlamydomonas* and *Synechocystis* (Schreiber et al. 1995) so extra attention should be paid to this effect when increasing the *spf* in quenching experiments and analysis on both plants and cyanobacteria.

### Adding a PSII_free_ component

So far we have considered only two different contributions. We now consider a third species: PSII_free_, i.e. PSII which is not coupled to any PB. Let *f*_2_ be its relative concentration. Expanding Eq. (3) the PAM signal of such a sample reads

6 $$J_{\text{PAM}} (I(t);{\text{PPc}}2,{\text{PB}}_{\text{free}} ,{\text{PSII}}_{\text{free}} ) = \left\{ {\begin{array}{*{20}c} {\gamma \times \phi_{{{\text{PB,}}u}} + c_{2} \times \phi_{{{\text{PPc2,}}o ,u}} + f_{2} \times \phi_{{{\text{PSII,}}o}} } \\ {\gamma \times \phi_{{{\text{PB,}}u}} + c_{2} \times \phi_{{{\text{PPc2,}}c ,u}} + f_{2} \times \phi_{{{\text{PSII,}}c}} } \\ \end{array} } \right.\,\,\,\,\,\,\begin{array}{*{20}c} {t \ne t_{\text{sat}} } \\ {t = t_{\text{sat}} } \\ \end{array} .$$

Two major differences should be noticed: first, the new species, PSII_free_, is spectrally characterized by the quantum yield *ϕ*_PSII_ which is not the same if the photosystem is attached to a PB antenna: *ϕ*_PPc2_ ≠ *ϕ*_PSII_. Second, the difference between the first and the second line in Eq. (6) lies in whether or not at a given time point *t* a saturation pulse was applied (denoted by *t*_sat_). If applied, such a pulse causes a shift in the PSII population which affects the quantum yield: for *t* = *t*_sat_, all PSII adopts a *closed state* (note the index *c* instead of *o*).

Just as in Eqs. (4) and (5), the contribution to the *F*_0,cyano_ and *F*_M,cyano_ in the case of these three components can be written as follows (see Table 1 for quantum yield values):

7 $$F_{{0,{\text{cyano}}}} = \gamma \times \phi_{{{\text{PB,}}u}} + c_{2} \times \phi_{{{\text{PPc2,}}o ,u}} + f_{2} \times \phi_{{{\text{PSII,}}o}} ,$$

8 $$F_{\text{M,cyano}} = \gamma \times \phi_{{{\text{PB,}}u}} + c_{2} \times \phi_{{{\text{PPc2}},c,u}} + f_{2} \times \phi_{{{\text{PSII}},c}}.$$

This time, Eqs. (7)–(8) build a system of *two* equations and *three* unknowns. This leads to *degeneracy* of the solutions, i.e. given this amount of information, more than only one set of parameters $c_{2} ,f_{2} ,\gamma$ are possible solutions of the system. A unique solution cannot be found unless additional conditions are introduced.

### NPQ

In the last section, we introduced a PAM model that is useful with two unquenched components only. However, during a PAM experiment, a second state, the quenched state, can be induced. A model of cyanobacterial NPQ is then needed if PAM measurements carried out under NPQ-inducing light conditions should be parameterized. A kinetic model of OCP-related NPQ has been developed by Gorbunov et al. (2011) in which a light-dependent reaction is responsible for the conversion of OCP^o^ to OCP^r^ which then can bind to the PB core and subsequently induce NPQ. A model reproducing the OCP^o^ → OCP^r^ conversion, the binding of OCP^r^ to PB and the FRP-related detaching of OCP is explained in detail in the SI [see “Derivation of the quenching and recovery dynamics” section in the Supporting Information (SI)]. A parameter *κ*_I_ describes how efficient the OCP^o^ → OCP^r^ conversion is (see also “Model applications” in SI). The initial concentration of [OCP^o^], hereafter expressed as a fraction of [PB], is estimated to be ca. 0.3–0.6 ([PB] = 1) in WT *Synechocystis*. The initial concentration [OCP^o^]_0_ and *κ*_I_ yield the time-dependent concentration of photoactivated OCP^r^. Decisive for NPQ induction is the encounter probability between PB and OCP^r^ [see Eq. (S11)]. The rate at which the binding occurs is *k*_1_, whereas *k*_2_ is the rate at which the FRP detaches OCP^r^ from the PB core (Gwizdala et al. 2013).

Under light conditions, the minimal, maximal and variable fluorescence are termed $F_{0}^{\prime}, \; F_{M}^{\prime} \; {\rm{and}} \; F_{V}^{\prime}$, respectively (see e.g. the review of Baker 2008). Our a priori knowledge of the fluorescence quantum yields of quenched states can help solving the problem of degeneracy: a set of analogous equations to Eqs. (7) and (8) (also shown below for clarity) can be postulated for the quenched state. Let *F*_0*,cyano*_^*q*^ and *F*_*M*,cyano_^*q*^ be the fluorescence levels of the *fully* quenched states; minimal and maximal fluorescence levels then read

7 $$F_{{0,{\text{cyano}}}} = \gamma \times \phi_{{{\text{PB,}}u}} + c_{2} \times \phi_{{{\text{PPc2,}}o ,u}} + f_{2} \times \phi_{{{\text{PSII,}}o}} ,$$

8 $$F_{\text{M,cyano}} = \gamma \times \phi_{{{\text{PB,}}u}} + c_{2} \times \phi_{{{\text{PPc2}},c,u}} + f_{2} \times \phi_{{{\text{PSII}},c}}.$$

9 $$F^{q}_{{0,{\text{cyano}}}} = \gamma \times \phi_{{{\text{PB,}}q}} + (1 - c_{0} ) \times \left( {c_{2} \times \phi_{{{\text{PPc2,}}o ,q}} + f_{2} \times \phi_{{{\text{PSII,}}o}} } \right) + c_{0} \times \left( {c_{2} \times \phi_{{{\text{PPc2,}}c ,q}} + f_{2} \times \phi_{{{\text{PSII,}}c}} } \right),$$

10 $$F^{q}_{\text{M,cyano}} = \gamma \times \phi_{{{\text{PB,}}q}} + c_{2} \times \phi_{{{\text{PPc2,}}c ,q}} + f_{2} \times \phi_{{{\text{PSII,}}c}} ,$$

where we have introduced *c*_0_ the fraction of PSII (free or bound) that is closed due to the high intensity of the NPQ-inducing light. Note that, in our model, only the species containing PB can be quenched; therefore, the PSII_free_ is not subject to NPQ during strong blue–green light exposure.

The additional condition introduced through the fully quenched state yields Eq. (10), which in combination with Eqs. (7) and (8) now builds a linear system of three equations with three unknowns. Alternatively, we can formulate the problem in a *n*_s_ **×** *m* matrix form *F* = **A** **×** *c*, where *F* and *c* are vectors and the matrix **A** has *n*_s_ rows corresponding to the *s* light-induced states of the sample and *m* columns associated with the number of fluorescent species:

11 $$\left( {\begin{array}{*{20}c} {F_{{0,{\text{cyano}}}} } \\ {F_{{M,{\text{cyano}}}} } \\ {F^{q'}_{{M,{\text{cyano}}}} } \\ \end{array} } \right) = \left( {\begin{array}{*{20}c} {\phi_{{{\text{PB,}}u}} } & {\phi_{{{\text{PPc2,}}o ,u}} } & {\phi_{{{\text{PSII,}}o}} } \\ {\phi_{{{\text{PB,}}u}} } & {\phi_{{{\text{PPc2,}}c ,u}} } & {\phi_{{{\text{PSII,}}c}} } \\ {\phi_{{{\text{PB,}}q}} } & {\phi_{{{\text{PPc2,}}c ,q}} } & {\phi_{{{\text{PSII,}}c}} } \\ \end{array} } \right) \times \left( {\begin{array}{*{20}c} \gamma \\ {c_{2} } \\ {f_{2} } \\ \end{array} } \right).$$

This system has a solution provided there is enough contrast in the fluorescence quantum yields between open and closed states [see Eqs. (S27), (S28)]. In that case, **A** is invertible and we obtain an estimate for the concentration vector:

12 $$\hat{c} = A^{ - 1} \times F.$$

Once the concentration vector has been estimated, Eq. (9) yields the estimated fraction of closed PPc2 and PSII_free_ when the high actinic light is turned on:

13 $$\hat{c}_{0} = \frac{{F^{q}_{{0,{\text{cyano}}}} + \hat{f}_{2} \times \left( {\phi_{{{\text{PPc2,}}o ,q}} - \phi_{{{\text{PSII,}}o}} } \right) + \left( {\hat{\gamma } - 1} \right) \times \phi_{{{\text{PPc2,}}o ,q}} - \hat{\gamma } \times \phi_{{{\text{PB,}}q}} }}{{\hat{f}_{2} \left( {\phi_{{{\text{PPc2,}}o ,q}} + \phi_{{{\text{PSII,}}c}} - \phi_{{{\text{PSII,}}o}} - \phi_{{{\text{PPc2,}}c ,q}} } \right) - \left( {\hat{\gamma } - 1} \right) \times \left( {\phi_{{{\text{PPc2,}}c ,q}} - \phi_{{{\text{PPc2,}}o ,q}} } \right)}}.$$

Finally, Fig. S10 shows the simulated data and the fit with the respective contributions resolved. The estimated parameters are given in Table S2 (trial Q1).

### Time evolution

**Fig. 3:**
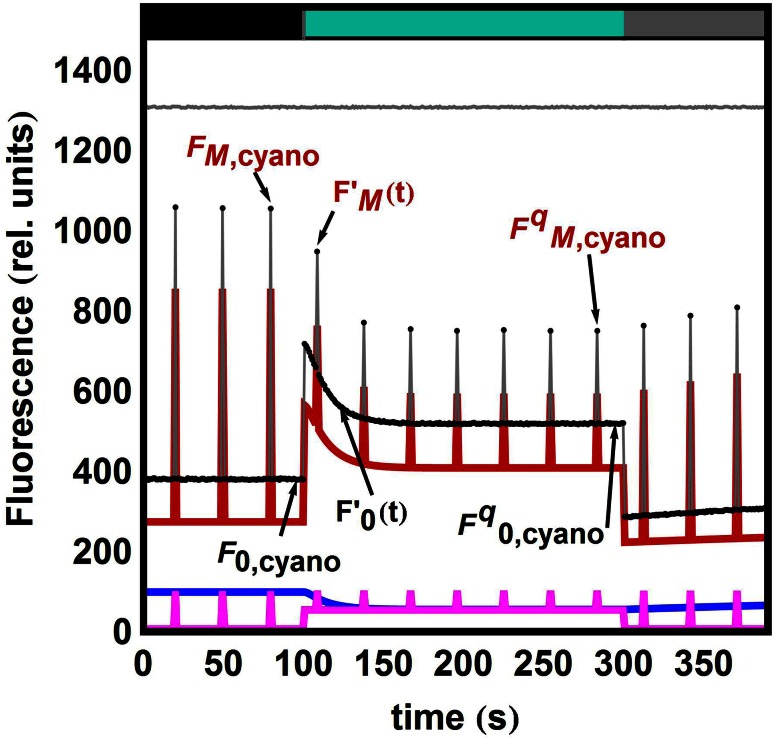
Simulated data with a pulse every 20 s and noise level of 0.01. At time *t* = 100 s, strong blue-green light is turned on (indicated by the *colour bars* on the top). Its power is such that a fraction *c*_0_ of the RCs are closed and the OCP^o^ → OCP^r^ conversion takes place with *κ*_I_ = 0.09 s^−1^. The amount of OCP^r^ formed is [OCP^r^] = 0.5 and it binds to PB with *k*_1_ = 0.30 s^−1^. The FRP detaches the OCP bound to the PB with *k*_2_ = 0.003 s^−1^. A fluorescence recovery region has been added: at time *t* = 300 s, the NPQ-inducing light is turned off. All RCs re-open and the OCP^o^ → OCP^r^ conversion stops (*κ*_I_ = 0). The action of FRP can be resolved better and *k*_2_ can be estimated (*k*_2_ = 0.00306 ± 0.00007 s^−1^). For all estimated parameters, see Table S2. *Black dots* simulated data points; *red* PPc2 contribution; *blue* PB_free_ contribution; *magenta* PSII_free_ contribution; *grey* sum of the three contributions. Residuals are shown on top with an offset of 1300

In the example discussed above, we have made use exclusively of the data points for which a static contribution could be given: Eqs. (7) and (8) correspond to the fully unquenched state, whereas Eqs. (9) and (10) describe the fully quenched state. In both cases, the respective concentrations do not vary in time. In a PAM experiment, however, one monitors a progressive time evolution from the unquenched to the quenched state following the introduced change in light conditions. Figure 3 shows an example: The first 100 s (grey bar on top indicates dim blue light regime) correspond to the static contributions. At *t* = 100 s, the actinic light is turned on (turquoise bar on top) and saturates partly the PSII reaction centres, shifting a fraction *c*_0_ of the PSII population from the open to the closed state (*F*_0_ becomes *F*_S_), regardless of the fact whether they are bound to a PB antenna or not. Concomitantly, the high actinic light triggers the photoconversion OCP^o^ → OCP^r^ and thus the formation of quenching complexes PB–OCP^r^, called *C*_q_ hereafter. *C*_q_ formation reflects in the overall decrease of fluorescence and the gradual shift within ca. 100 s towards another state characterized by the fluorescence quantum yields of the fully quenched species (*t* > 200 s) which can be satisfactorily described by either Eq. (9) or (10). Suppose we would like to know how the maximal fluorescence from the complex PPc2 evolves from the ‘unquenched’-static level described by $c_{2} \cdot \phi_{PPc2,c,u}$ to the ‘quenched’-static level described by $c_{2} \cdot \phi_{PPc2,c,q}$. What we are looking for is thus an adequate expression for *c*_2_(*t*).

Let $F_{\rm s}^{\prime}$(*t*) and $F_{\rm M}^{\prime}$(*t*) be the respective levels of steady-state and maximal fluorescence at any given time *t* under strong blue–green illumination. The data points within the interval 100 < *t* < 200 s in Fig. 3 show a time-evolving mixture of quenched and unquenched species, i.e. the expression we are interested in necessarily has the mixed form (consider, for simplicity, only the maximum fluorescence):

14 $$F_{\text{M,PPc2}} ' (t )= c_{2,u} (t )\times \phi_{{{\text{PPc2,}}c ,u}} + c_{2,q} (t )\times \phi_{{{\text{PPc2,}}c ,q}} ,$$

where of course, the sum of quenched and unquenched species yields the total amount of complexes: *c*_2,*u*_(*t*) + *c*_2,*q*_(*t*) = *c*_2_. The equation system we need to solve to determine the time-dependence is (see “Derivation of the quenching and recovery dynamics” in SI):

15 $$PB'(t) = - k_{1} \times {\text{PB}}^{2} (t) + \left( {k_{1} \times \left( {{\text{PB}}_{0} - {\text{OCP}}^{\text{r}}_{0} - {\text{OCP}}^{\text{o}}_{0} + {\text{OCP}}^{\text{o}} (t)} \right) - k_{2} } \right) \times {\text{PB}}(t) + k_{2} \times (C_{q,0} + {\text{PB}}_{0} ),$$

16 $${\text{OCP}}^{{{\text{o}}'}} (t) = - \kappa_{\text{I}} \times {\text{OCP}}^{\text{o}} (t) + k_{3} \times \left( {{\text{PB}}(t) - {\text{PB}}_{0} + {\text{OCP}}_{0}^{\text{r}} + {\text{OCP}}_{0}^{\text{o}} - {\text{OCP}}^{\text{o}} (t)} \right).$$

We call the set of numerical solutions to this system *Q*_*i*_(***P***,*t*) where the index *i* indicates which of the four concentration profiles the quenching function describes. We use *u* for unquenched PB, *q* for *C*_q_, o for the orange form of OCP and r for its red form. The time-dependence can be easily expressed as the product of an initial concentration value *c*_*j*_ and the corresponding profile given by *Q*_*i*_(***P***,*t*). Furthermore, recall that the kinetic rates are defined as *κ*_I_ light-intensity-related activation of OCP^o^, *k*_1_ OCP-binding rate, *k*_2_ FRP-related detaching rate, and *k*_3_ deactivation of OCP^r^. Thus, the set of parameters read: ***P*** = ([*PB*], [*OCP*^o^], [*OCP*^r^], [*C*_*q*_], *k*_1_,*k*_2_,*k*_3_,*κ*_I_). We include in the SI several graphical solutions of *Q*_*i*_(***P***,*t*) evaluated with different arguments (Figs. S5, S6).

Let the vector $P^{0} = ( {[ {PB}]^{0},\,[ {OCP^{\text{o}} }]^{0},\,[ {OCP^{\text{r}} } ]^{0},\,[ {C_{q} }]^{0},\,k_{1}^{0},\,k_{2}^{0},\,k_{3}^{0},\,\kappa_{\text{I}}^{0} })$ be a vector containing a defined set of parameters for which we obtain the function *Q*_*i*_(***P***^**0**^,*t*). The time-dependent expression [given in Eq. (14)] for the maximal fluorescence *F*_M_′(*t*) resulting from the PPc2 species at any given time *t* within the dynamic region 100 < *t* < 200 s would then read

17 $$F_{\text{M,PPc2}} ' (t )= c_{2} \times Q_{u} (P^{ 0} ,t )\times \phi_{{{\text{PPc2,}}c ,u}} + c_{2} \times Q_{q} (P^{ 0} ,t )\times \phi_{{{\text{PPc2,}}c ,q}} .$$

Therefore, the whole expression for $F_{\rm M}^{\prime}$(t) in this three-component example is given by

18 $$F_{\text{M}}^{\prime} (t )= \gamma \times \left( {Q_{u} (P^{ 0} ,t )\times \phi_{{{\text{PB,}}u}} + Q_{q} (P^{ 0} ,t )\times \phi_{{{\text{PB,}}q}} } \right) + c_{2} \times \left( {Q_{u} (P^{ 0} ,t )\times \phi_{{{\text{PPc2,}}c ,u}} + Q_{q} (P^{ 0} ,t )\times \phi_{{{\text{PPc2,}}c ,q}} } \right) + f_{2} \times \phi_{{{\text{PSII,}}c}} .$$

An analogous equation can be written for the time-dependent Eq. (9).

Modelling the time evolution also helps clarifying the following: a *fully* quenched state [see Eq. (10)] does *not* mean that all the PBs present in the sample are quenched; rather the fraction of OCP that gets activated dynamically equilibrates the system in this *fully* quenched state. Notice Fig. S8 in the SI, in which it is illustrated that an increasing OCP/PB ratio affects the equilibrium level at *t* *→* *∞*.

### Estimating OCP-related parameters

In addition to resolving the different species-related contributions, we also gain insight into NPQ kinetics. Since there is a gradual equilibration between the unquenched and the quenched state, the data points in the region *t* > 100 s of Fig. 3 deliver information over the rates *k*_1_, *k*_2_ and the amount of OCP^o^ present in the cells. Table S2 contains estimated parameters in different trials. Trials Q1–Q4 (Q indicating that next to the *F*_0_ region, only quenching region data are used, but not the recovery region) were performed on the simulated data shown in Fig. S10. In trial Q1, no kinetic parameters were freed; in trial Q2, we free the OCP^r^–PB binding rate *k*_1_. Note that the *t* values slightly change for all the estimated parameters, but in principle, all of them have been estimated reliably. This does not hold true for trials Q3 (free parameters: *k*_1_ and *k*_2_) and Q4 (free parameters: *k*_1_, *k*_2_ and OCP_0_^o^) where we stepwise increase the number of parameters we want to estimate. In particular, the reliability with which the FRP-related detaching rate *k*_2_ is estimated drastically drops in these two last trials. The conclusion is, therefore, that there is not enough information to estimate all of the seven parameters accurately, the detaching rate *k*_2_ being the most challenging to estimate correctly.

In order to add valuable information that allows all parameters, including *k*_2_, to be estimated, we simulate a new region for *t* > 300 s where the sample is exposed to dim blue light (grey bar on top) and *κ*_I_ becomes negligible (see Fig. 3). In this light regime, the amount of formed OCP^r^ is negligible and the quenching complexes are no longer formed. Hence, a region of fluorescence recovery delivers additional information (trial *Q* + *R*) so that also *k*_2_ can be estimated reliably (see Table S2 for *t* values).

### Adding PSI_free_ as a third component

Above we have chosen the PSII_free_ contribution to illustrate the problem of degeneracy. Instead, we could have chosen any other realistic contribution, for instance, a PSI-related contribution. Due to the greater number of PSI than PSII in *Synechocystis* (Moal and Lagoutte 2012), the PSI contribution, despite the short PSI fluorescence lifetime, is not negligible. Let the concentration of free PSI trimers, *f*_1_, be *β* times greater than the PSII-dimer concentration *c*_2_. Furthermore, we will simplify the problem by considering PB to be a PSII-specific antenna, i.e. no PSI complex has any PB attached to it (PSI_free_). Analogous equations to (7)–(10) can then be written (see “The PSI contribution” in the SI). Notice that, in Eq. (S30), PSI is assumed not to adopt any closed or open states or to undergo quenching (in this example, PB does not attach to PSI). Figure S11 shows the simulated data and the fit with the PB_free_, PPc2 and PSI_free_ contributions, respectively, resolved. The constant PSI_free_ contribution, present in all conditions, correlates strongly with the *scale* and NPQ parameters and cannot be accurately estimated due to numerical unidentifiability. Hence, it is necessary to make further assumptions based on independent measurements in order to obtain interpretable results.

### A case study

**Fig. 4:**
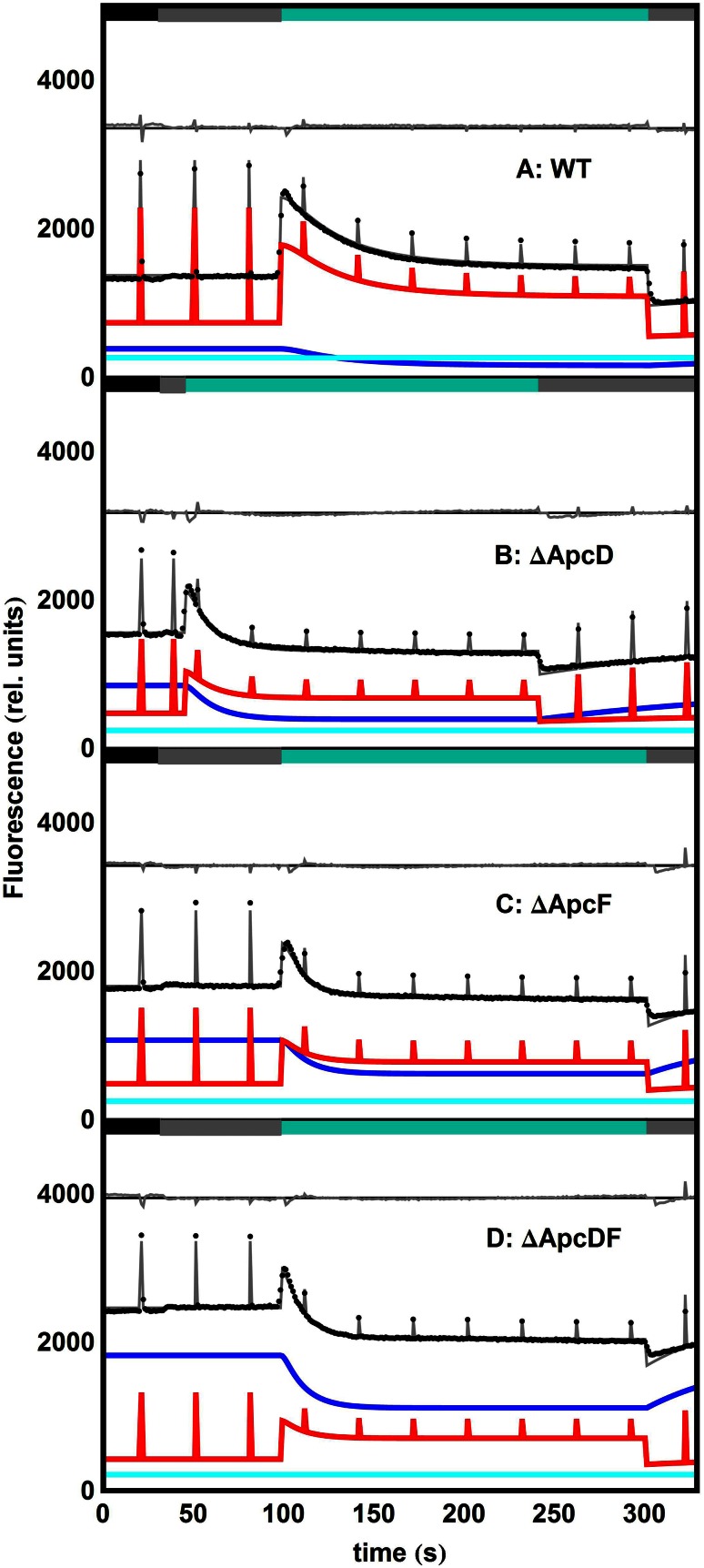
Fit of experimental PAM measurements performed on whole cells of WT *Synechocystis* (**a**) and mutants thereof: ∆ApcD (**b**), ∆ApcF (**c**), ∆ApcDF (**d**). *Colour bars* on the top of each panel indicate the light regime: dark (*black*), 30 µE (*grey*) and 1400 µE (*turquoise*). All estimated parameters are collated in Table 2. *Black dots* experimental data points; *red* PPc2 contribution; *blue* PB_free_ contribution; *cyan* PSI_free_ contribution; *grey* sum of all contributions. Residuals (*grey*) are shown in each panel with an offset

Figure 4a shows experimental data from a PAM experiment carried out on whole cells of wild-type *Synechocystis* (Jallet et al. 2012) that has been fitted using our model with the following components: PPc2, PB_free_ and PSI_free_. After analysis, the PSII_free_ contribution of these samples turned out to be negligible. The PSI/PSII proportionality factor *β* has been set to *β* = 3.6 based on *Synechocystis* stoichiometry published by Moal and Lagoutte (2012). The grey curve fits the data points (shown in black). This grey curve is the sum of all other contributions: the PPc2 in red, the PSI_free_ in cyan and PB_free_ in blue. Residuals are shown in the upper part of the figure. The estimated parameters are shown in Table 2 and in more detail in Table S3. Notice that, during the experiment, the light intensity is manually adjusted: turning the strong blue light on and off is modelled as being instantaneous and therefore it cannot possibly describe the data accurately in the transition regions. For this reason, the contributions to the error analysis of the data points lying in the respective transition regions have been arbitrarily weighted down to one tenth.

**Table 2:** Estimated parameters from experimental PAM measurements performed on whole cells of WT *Synechocystis* and mutants thereof 21

	WT	∆ApcD	∆ApcF	∆ApcDF
*γ*	**0.034**	**0.059**	**0.072**	**0.131**
*c*_0_	0.606	0.564	0.571	0.577
*scale*	13899.6	14314.8	14838.5	13942.9
*k*_1_	**0.121**	**0.243**	**0.302**	**0.451**
*k*_2_	**0.0096**	**0.0068**	**0.019**	**0.018**
OCP_0_^o^	0.61	0.64	0.53	0.46

The amount of PSI_free_ has been fixed (see Table S3). Bold parameters indicate the most important results

In addition to WT, mutants that lack the core terminal emitter subunits allophycocyanin D (ΔApcD) or allophycocyanin F (ΔApcF), or both (ΔApcDF) have been measured. The difference in free energy between PB and PSII is smaller in WT than in the mutants that lack APC680 pigments. However, we assume the same set of quantum yields (Table 1). This implies that PB either transfers to PS with a certain rate or not (PB_free_). Target analysis of streak camera measurements is needed to test this assumption.

The results for the mutants ΔApcD, ΔApcF and ΔApcDF are shown in Fig. 4b–d. The relative amount of PB_free_ in each sample increased in the order WT → ΔApcD → ΔApcF → ΔApcDF. This rationalizes two observations: first, the *F*_0_-level also increases in the same order even though the amount of Chl *a* remained the same. This means that the mutations all affected the energy transfer to the RCs just as observed by Bryant (1991) and Dong et al. (2009). And second, after 200 s of intense blue–green illumination, the steady-state fluorescence *F*′_s_(*t*) in the mutants is quenched below *F*_0,cyano_ in dim blue light. Indeed, this can only be explained by means of a large amount of quenched uncoupled PB.

**Fig. 5:**
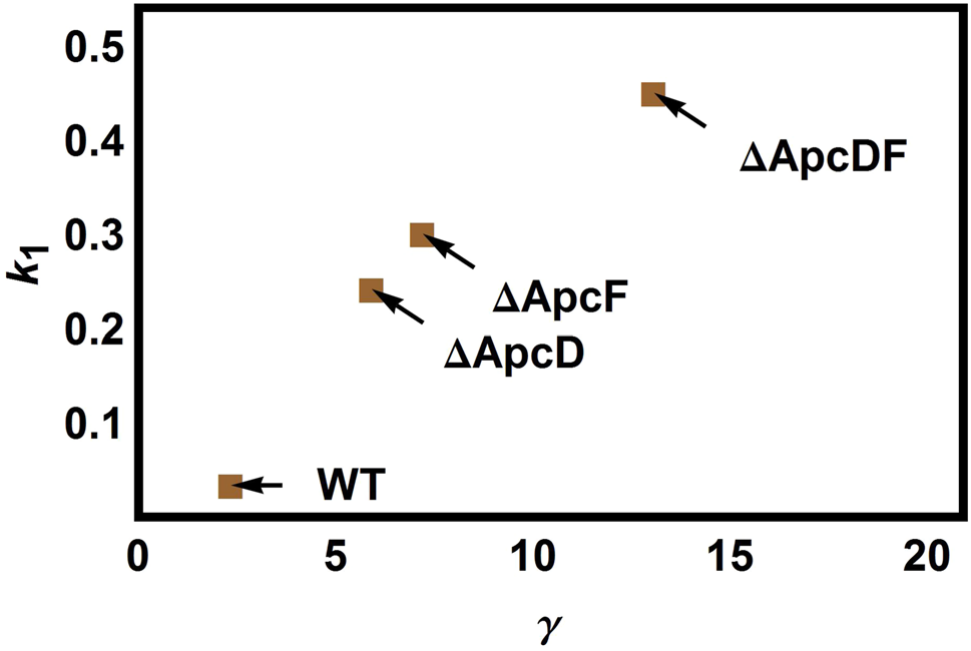
Correlation between the estimated parameters γ and *k*_1_ in the different samples

The parameter related to fluorescence recovery, *k*_2_, seems not to be affected by an increasing amount of PB_free_ but by the presence (or absence) of ApcF. Both WT and the ΔApcD mutants have indeed similarly slower fluorescence recovery rates than the two mutants that lack ApcF. In contrast, *k*_1_ does increase in the order: WT → ΔApcD → ΔApcF → ΔApcDF suggesting that OCP gets quicker attached to the PB core. Thus, the lack of the ApcD or ApcF could affect the ability of the PB to strongly bind to the membrane and to efficiently functionally couple to the photosystems resulting therefore in a more important amount of PB_free_ (see definition in ‘A model to resolve several contributions to a cyanobacterial PAM-signal’) in single-mutated systems than in WT, the greatest amount of PB_free_ being that of the double mutant ΔApcDF. Figure 5 depicts the correlation between PB_free_ and *k*_1_. It would be indeed consistent with the idea that the less tightly bound the PB is to the membrane, the easier it gets for external proteins just like OCP and FRP to reach the binding site.

An alternative interpretation is that the mutations affect the rate of energy transfer from PB to PSII. In that case, a different set of quantum yields (cf. Table 1) for each of the mutants will be needed, based upon target analysis of streak camera measurements. Such measurements can also demonstrate the presence of increased amounts of uncoupled PB with a 1.6 ns lifetime. These measurements and analyses are in progress. Further information can be gained from spectrally resolved fluorescence induction (Kana et al. 2012; Kirilovsky et al. 2014).

Note that the residuals of all samples are acceptable except for one systematic pattern: during the very first seconds of the measurement, the fluorescence is collected in darkness (black bar on top of each panel). *Synechocystis* cells are known to be in state 2, where PSI gets more excitation energy. The transition from darkness to dim blue light draws a characteristic feature in the PAM trace: the relative distribution of excitation energy from the PB to both of the photosystems changes and more of the energy will be transferred to PSII (state 1) hence why a subtle increase in *F*_M_ and also in *F*_0_ is observed (*F*_0_ also varies due to a small fraction of photosystems being closed by the dim blue light). Since our model does not include this relative change that affects the concentration *c*_2_, it systematically fails to match both *F*_M_ and the *F*_0_ levels. The best fit is the one of the mutant ΔApcD—a mutant for which state transitions have been reported to be severely impaired (Dong et al. 2009) and can therefore be modelled somewhat better by a model that implicitly assumes the cells to be locked in one state.

## Concluding remarks

We have presented a model for cyanobacterial PAM-traces taking into account the phycobilin-related contribution. The main feature of our model is that several fluorescent species can be resolved and their concentrations be estimated. This is remarkable because the traditional use of a PAM experiment has been to determine the dynamic changes of fluorescence quantum yields of species whose concentrations were previously known. Besides, this new approach opens up the possibility to the analysis of quenching curves in cyanobacteria making use of the knowledge already developed for PAM signals in plants.

The energy-dependent component of NPQ in cyanobacteria has also been modelled. This allowed typical quenching curves of experiments tracking fluorescence carried out in vitro and in vivo to be parameterized and analysed. The full dynamics could be reproduced: the fast opening and closing dynamics of RCs due to short saturation pulses, OCP-driven NPQ and FRP-assisted fluorescence recovery kinetics. Our model was used to quantify OCP-related parameters while resolving the different components and determining their time-dependent concentrations in the sample. Subsequently, the following conclusions have been drawn: ApcD and ApcF play a role in tightly binding the PB to the thylakoid membrane. Mutants where these pigments were missing contained ca. 3 % more functionally uncoupled phycobilisomes. Besides, the estimated OCP-attaching (and detaching) rates consistently vary from mutant to mutant providing these experimental results with a model-based interpretation. However, the interpretation suggested by these results does not exclude the scenario where PBs lacking ApcD and ApcF transfer energy less efficiently to the PS.

This model can be used for systematically assessing the emissive species’ content in samples that lack any of the (or both) photosystems, have been grown under different light conditions or even those which have undergone targeted stress, i.e. iron or copper deprivation. In a broader context, our model is a potential tool for all cyanobacterial photosynthesis studies: from basic research in photosynthesis to environmental studies in which cyanobacterial populations in seas or lakes are monitored using a PAM instrument.

## Electronic supplementary material

Supplementary material 1 (DOCX 506 kb)

## Abbreviations

APC: Allophycocyanin
Chl: Chlorophyll
C-PC: C-phycocyanin
FRP: Fluorescence recovery protein
NPQ: Non-photochemical quenching
OCP: Orange carotenoid protein
PAM: Pulse-amplitude modulated
PB: Phycobilisome
PC: Phycocyanin
PPc2: Phycobilisome–Photosystem II complex
PQ: Plastoquinone
PSI: Photosystem I
PSII: Photosystem II
rmse: Root mean square error
SNR: Signal-to-noise-ratio
