# Supplementary material for: Resolving the contribution of the uncoupled phycobilisomes to cyanobacterial pulse-amplitude modulated (PAM) fluorometry signals
Source: Photosynth Res. 2015 Apr 19;127(1):91–102. doi: 10.1007/s11120-015-0141-x (PMC4673099; doi:10.1007/s11120-015-0141-x)
Supplement: Supplementary file 1 — Supplementary material 1 (DOCX 506 kb) [file 11120_2015_141_MOESM1_ESM.docx]

# Supporting Information


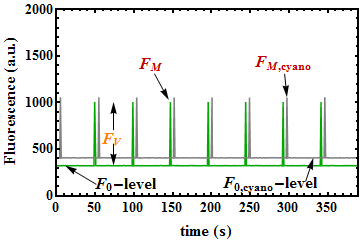


Figure S 1 Simulated PAM–signal of *i)* dark–adapted PSII as it is expected in plants (green) and *ii)* PSII and with an extra phycobilin contribution as it is expected in cyanobacteria (gray). A saturation pulse has been applied every 50 s (dephased for visibility).


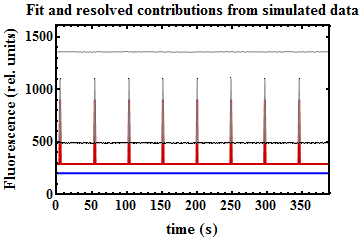

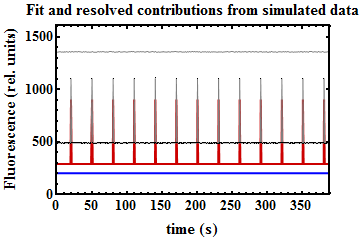


Figure S 2 Simulated data with noise level 0.01 and a pulse every 50 s (left panel) and 20 s (right panel). These datasets have been fitted with the model containing two components: PPc2 and PB_free_. Key: Black dots: simulated data points; Red: PPc2-contribution; Blue: PB_free_-contribution; Gray: sum of the two contributions. Residuals are shown on top with an offset for better visibility.

## On the relationship between Chl a fluorescence and photochemistry.

Three essential assumptions are made in order to use fluorescence as a proxy for photosynthetic activity. First, harvested light has three possible pathways: either it reaches the reaction centers of PSII where it is used for photochemistry (_ph_) or it gives rise to fluorescence (_fl_) or it gets dissipated as heat (_h_):

|  |  | (S1) |
| --- | --- | --- |

Second, a saturation–pulse induces a state where Q_A_ is reduced, *i.e.* transient saturation of PSII (closed centers), while the fluorescence and heat dissipation yields are maximal:

|  |  | (S2) |
| --- | --- | --- |

And last, the ratio of the fluorescence and heat dissipation yields is assumed not to vary during the saturating pulse:

|  |  | (S3) |
| --- | --- | --- |

Therefore, combining Eq. (S2) and (S3) we obtain an expression for (_h_)_M_ that can be replaced in Eq. (S1) so that the yield of photochemistry is strictly related to the one of fluorescence:

|  |  | (S4) |
| --- | --- | --- |

This relationship has propelled numerous studies aiming to track fluorescence under controlled light conditions and relate those measurements to the PSII photochemical yield. The parameter:

|  |  | (S5) |
| --- | --- | --- |

directly derived from Eq.(S4), has been commonly equaled to the index of maximal photochemical efficiency of PSII in plant studies. We therefore obtain (see Eqs. & ):

|  |  | (S6) |
| --- | --- | --- |

Hence, for a fixed level of Chl *a* (PSII) fluorescence, the cyanobacterial η_cyano_ decreases with increasing phycobilisome/Chl ratio or disconnected PBs/PSII (for an example under Fe starvation see Wilson et al. 2007). illustrates Eq. (S6). Note that this decrease is *not* related to a change in photochemistry. Thus, without knowing how much the PB contributes to the signal, the parameter F_V_/F_M_ cannot be used to measure the maximal photochemical efficiency of PSII. In addition, when performed in plants, these measurements are done in darkness, conditions in which there is no NPQ. In contrast, cyanobacterial cells tend to state 2 in darkness Campbell et al. 1998; as a consequence F_M_ is quenched.


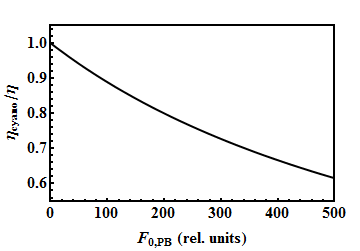


Figure S 3 Normalized expression for cyanobacterial η_cyano_ (with respect to plants) as a function of the phycobilin-related contribution F_0,PB_.

Using Eq. we can re-write Eq.(S6):

|  |  | (S7) |
| --- | --- | --- |

with:

|  |  | (S8) |
| --- | --- | --- |


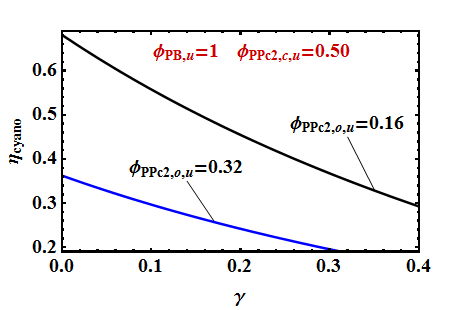


Figure S 4 η_cyano_ as a function of the relative concentration of PB_free_ and the difference of PPc2 quantum yields in open and closed states. Two curves have been plotted for two distinct values of φ_PPc2,o,u_ while φ_PPc2,c,u_ has been kept constant. The graph illustrates how the variable fluorescence decreases with decreasing contrast between open and closed states: _PSII_=φ_PPc2,c,u_ – φ_PPc2,o,u_. Key: Black: _PSII_=0.34; Blue: _PSII_=0.18.

## Derivation of the quenching and recovery dynamics.

Formation of a quenching complex C_q_. The homogeneous system**.** The system of second order differential equations describing the formation of a quenching complex *C_q_* from an activated OCP^r^ that binds to a PB is:

|  |  | (S9) |
| --- | --- | --- |

|  |  | (S10) |
| --- | --- | --- |

|  |  | (S11) |
| --- | --- | --- |

where the product PB(t)·OCP^r^(t) accounts for the encounter probability between a photoactivated OCP and a PB.

Note that PB’(t) = OCP^r^’(t) = – C_q_’(t). This property allows the system to be simplified and expressed in terms of the single variable PB(t).

After subtraction of Eq. (S9) from Eq. (S10) we obtain the homogeneous equation:

|  |  | (S12) |
| --- | --- | --- |

from which it follows that the general solution is:

|  |  | (S13) |
| --- | --- | --- |

where *a*_1_ is an arbitrary constant. We obtain the value of *a*_1_ by simply evaluating both functions at time t=0. Thus:

|  |  | (S14) |
| --- | --- | --- |

Replacing Eq. (S14) in Eq. (S13) yields an expression of OCP^r^(t) as a function of PB(t) given some starting values for the PB and photoactivated form of OCP at time t=0:

|  |  | (S15) |
| --- | --- | --- |

Furthermore, adding Eq. (S9) to Eq. (S10) leads to a row of equations similar to Eqs.(S11)–(S15) in the case of the quenching complex *Cq*(t). We obtain the final expression:

|  |  | (S16) |
| --- | --- | --- |

By inserting Eqs. (S15) & (S16) in Eq. (S11) we finally obtain a single equation of second order for the single variable PB(t). Its graphical solution is shown in Figure S 5A :

|  |  | (S17) |
| --- | --- | --- |

| **A: Parameters:**  ***k_1_=*0.025; *k_2_=*0.001; OCP^r^_0_=0.5; OCP^o^_0_=0**  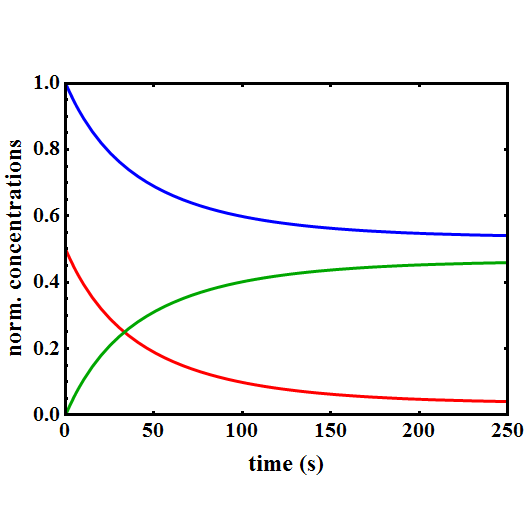 | **B: Parameters:**  ***k_1_=*0.025; *k_2_=*0.001; OCP^r^_0_=0; OCP^o^_0_=0.5**  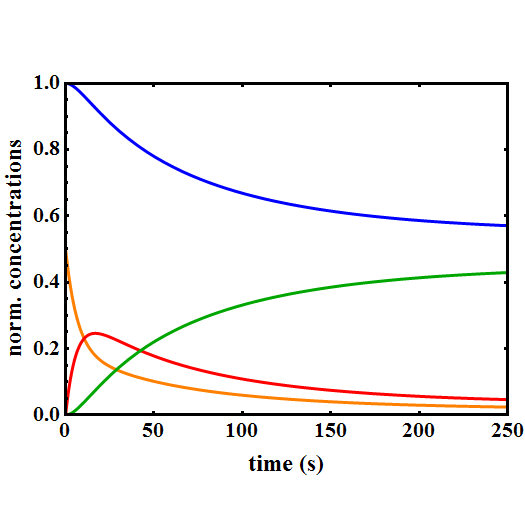 |
| --- | --- |
| **C: Parameters:**  ***k_1_=*0.025; *k_2_=*0.01; OCP^r^_0_=0; OCP^o^_0_=0.5**  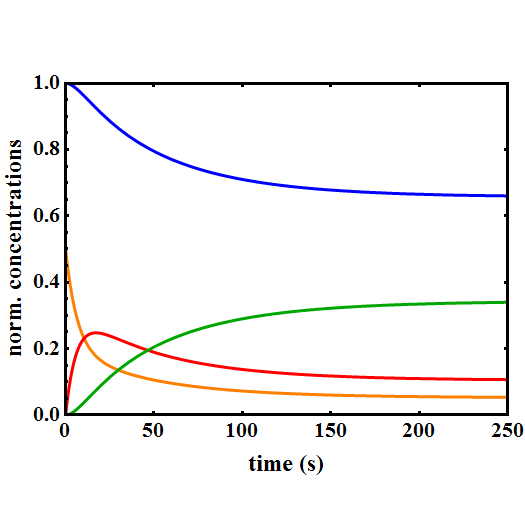 | **D: Parameters:**  ***k_1_=*0.025; *k_2_=*0.001; OCP^r^_0_=0; OCP^o^_0_=0.33**  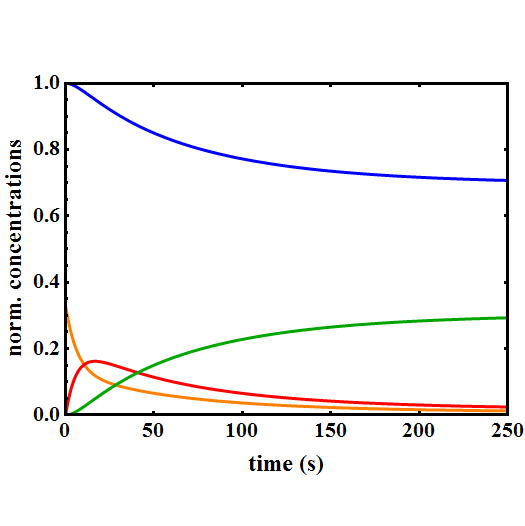 |

Figure S 5 Graphical solutions shown for a homogeneous (A) and an inhomogeneous (B–D) system of differential equations reproducing the NPQ-dynamics over time. In all panels: [PB_0_]=1, [*C_q_*]=0. In panels B–D: _I_=0.1, *k_3_*=0.05. Key: blue: *Q_u_*; red: *Q_r_*; green: *Q_q_*; orange: *Q_o_* (see text for explanation).

The light-intensity dependent generation of OCP^r^. The inhomogeneous system**.** The observed kinetics up to now imply a non–zero concentration of OCP^r^ at time t=0 (note OCP^r^_0_=0.5 in Figure S 5A). This is, however, far from the experimental observation: OCP has to be photo–converted by light absorption from its orange to its red form first. This process depends on the wavelength and intensity of the light.

The time–dependent generation of OCP^r^ from the orange form of a fixed amount of OCP^o^ will therefore be introduced as an inhomogeneity in Eq. (S12) with _I_ being the light-intensity dependent formation rate of OCP^r^:

|  |  | (S18) |
| --- | --- | --- |

which once integrated and evaluated at time *t*=0 (for the left side of the equation we know the answer already from Eq. (S13)):

|  |  | (S19) |
| --- | --- | --- |

yields the constant *a_2_* = *a_1_* + OCP^o^_0_ and we obtain thus the solution for all times *t*:

|  |  | (S20) |
| --- | --- | --- |

An analogous equation to Eq. (S17) can now therefore be derived by inserting Eq. (S20) instead of Eq. (S15) in Eq. (S9) (see Eq. (S21)).

Finally, we introduce a last kinetic parameter, namely a deactivation rate of OCP^r^ *k_3_* that accounts for the fact that the population OCP^o^ grows as OCP^r^ deactivates. The system of differential equations we need to solve reads:

|  |  | (S21) |
| --- | --- | --- |

|  |  | (S22) |
| --- | --- | --- |

A solution to this system can only be given numerically. The graphical solution of this quenching function which we call *Q_i_(****P****,t)* is shown in for a chosen set ***P*** of parameters. *Q_i_(****P****,t)* has four components: *Q_u_* (blue) describes de unquenched PB, *Q_q_* (green) the quenching complex *C_q_*, *Q_o_* (orange) the orange form of OCP and *Q_r_* its photo–converted red form. Notice that this time OCP^r^_0_=0 while OCP^o^_0_=0.5. The deactivation rate of OCP^r^ *k_3_* has been chosen to be smaller (*k_3_*=0.05) than the activation rate (κ_I_=0.1).

The recovery phase**.** Turning off the actinic light simply translates in setting the parameter _I_ to zero. Furthermore, we assume that during the quenching phase only a fraction of the orange OCP is converted to its red form. The graphical solution of the recovery phase with two different sets of parameters (*k_2_* is varied) is shown in .

| **A: Parameters:**  **_I_*=*0; *k_2_=*0.001**  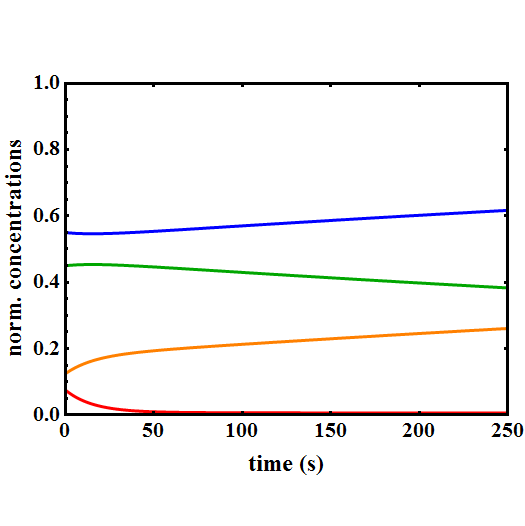 | **B: Parameters:**  **_I_*=*0; *k_2_=*0.005**  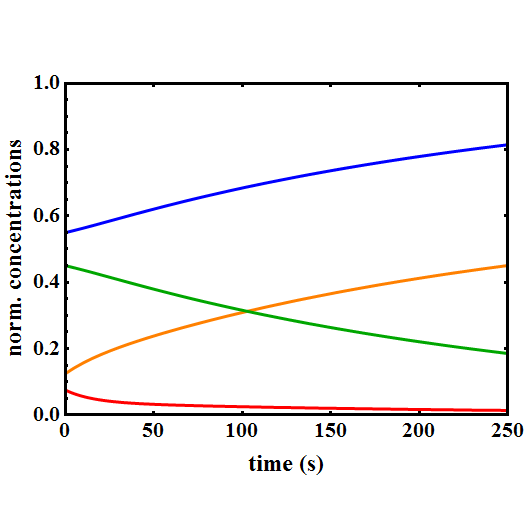 |
| --- | --- |

Figure S 6 Graphical solution for the recovery dynamics (κ_I_=0) with *k_2_*=0.001 (A) and *k_2_*=0.005 (B). Parameters: PB_0_=0.55; *C_q,0_*=0.45; OCP^r^_0_=0.1; OCP^o^_0_=0.001; *k_1_*=0.025; *k_3_*=0.05. Key: blue: *Q_u_*; red: *Q_r_*; green: *Q_q_*; orange: *Q_o_*

# NPQ Model applications

## Light-dependent OCP^o^→OCP^r^ conversion.

The NPQ dynamics in the cyanobacterium *Synechocystis* has been studied by Gorbunov et al. 2011. In Fig. 3B of their publication they show how NPQ is differently induced when varying the ambient light intensity. We have digitized their data using Plot Digitizer, an open source tool to create readable files from graphs. The digitized data has then been fitted with our model (see left panel of ). The light–intensity dependent rate is *κ_I_*, i.e. the rate with which the OCP intermediate state is formed in the Gorbunov’s model. Their result shows that the rate of NPQ induction accelerates with an increase in photon flux density of actinic blue light Gorbunov et al. 2011. We have modelled the same effect by choosing a set of realistic starting values. Two parameters have been estimated: the OCP^o^→ OCP^r^ conversion rate *κ_I_* and the initial concentration of OCP in its orange form, [OCP^o^].

Table S 1 shows the results. The initial concentration [OCP^o^] is in the vicinity of 60% ([PB]=100%) for all the samples. The behaviour of *κ_I_* as a function of the light intensityis the one expected: the more intense the ambient actinic light, the quicker the photoactivation of OCP, and therefore, the quenching process. A scatter plot relates the estimated *κ_I_* to the light intensity value (right panel in ). In principle, the model can be used to find adequate *κ_I_* values given an unknown ambient actinic light intensity via a simple linear regression.

| 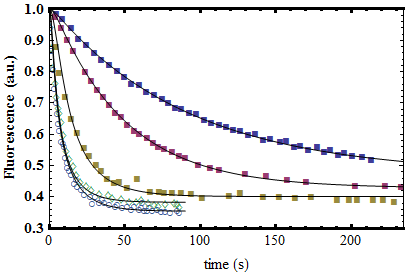 | 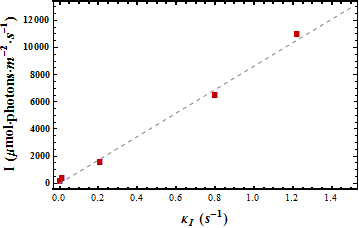 |
| --- | --- |

Figure S 7 *Left:* Light intensity dependence of NPQ induction as studied by Gorbunov, *et al.* Key: 200 (blue closed squares), 400 (magenta closed squares), 1600 (yellow closed squares), 6500 (green open diamonds) and 11000 (blue open circles) μmol·m^-2^·s^-1^. Fit curves are shown in black. *Right:* Scatter plot displaying the conversion rates _I_ as estimated from the fits against ambient actinic light’s intensity as given by Gorbunov, *et al.* A linear regression is also shown in gray.

| I (μE) | 200 | 400 | 1600 | 6500 | 11000 |
| --- | --- | --- | --- | --- | --- |
| [OCP^o^] | 0.54 | 0.58 | 0.64 | 0.64 | 0.68 |
| _I_ | 0.01 | 0.02 | 0.22 | 0.81 | 1.23 |

Table S 1 Estimated parameters applying our model to the digitized data of Gorbunov, *et al*.

## Estimating the OCP/PB ratio of in vitro experiments.

Our model was also able to reproduce fluorescence decrease as first observed *in vitro* by Gwizdala et al. 2011 and published in Jallet et al. 2012. NPQ was induced by blue–green light of 900 μmol·m^-2^·s^-1^ (left panel in ) in samples containing a phosphate concentration of 0.5 M, where the ratio OCP/PB was varied (4, 8, 20 and 40). Since these experiments were carried out *in vitro* some of the kinetic parameters differ slightly from the Gorbunov example discussed above. The data was satisfactorily fitted and the different ratios OCP/PB (shown next to the curves in ) were estimated. They agree surprisingly well with the experimental conditions chosen by Jallet, *et al.*, the greatest error being of ca. 10% in the data point OCP/PB*^(exp)^* = 40 (OCP/PB*^(est)^* = 51).

Jallet, *et al.* carried out an additional experiment with the phosphate concentration being 0.8 M. Even though the model does not include any *buffer parameter* and it is far from predicting any conformational changes, we assumed that these effects would ultimately translate in affected OCP binding/detaching rates. Thus, we were still able to fit the data () and extract again the ratios OCP/PB*^(est)^*. The results agreed fairly well with the experimental results.

| 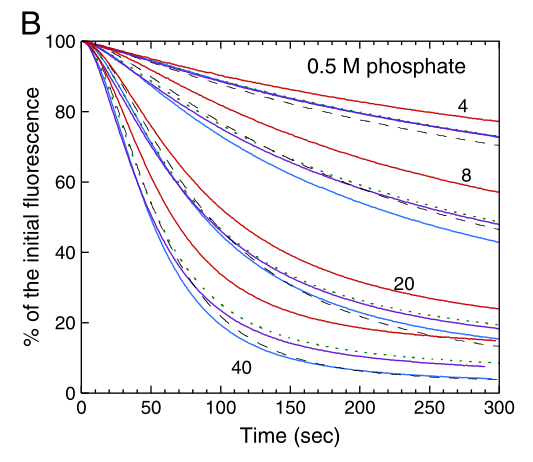 | 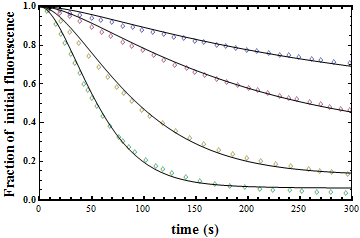  **51.5**  **23.8**  **7.9**  **3.7** |
| --- | --- |

Figure S 8 *Left:* Fluorescence decrease induced with blue-green light (900 μmol·m^-2^·s^-1^) with different OCP to PBs ratios (4, 8, 20 and 40) in samples containing 0.5 M Phosphate as published by Jallet, et al. (2012). *Right:* Digitized wild type data (black dashed curves on the left panel) fitted using our NPQ model. The only parameter varying from curve to curve is the initial concentration [OCP^o^] which results in different OCP to PBs ratios. The estimated ratios are shown next to the corresponding fit.

| 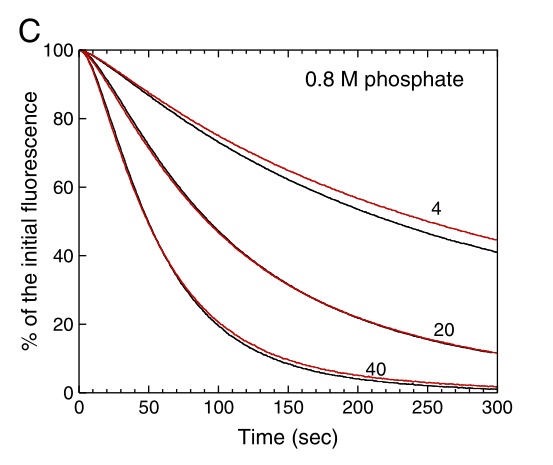 | 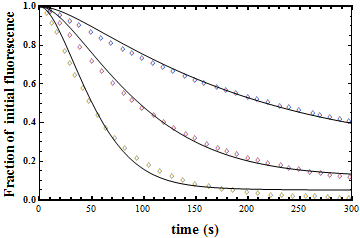  **6.9**  **17.0**  **41.5** |
| --- | --- |

Figure S 9 *Left:* Fluorescence decrease induced with blue-green light (900 μmol·m^-2^·s^-1^) with different OCP to PBs ratios (4, 20 and 40) in samples containing 0.8 M Phosphate as published by Jallet, et al. (2012). *Right:* Digitized data fitted using our NPQ model. The only parameter varying from curve to curve is the initial concentration [OCP^o^] which results in different OCP to PBs ratios. The estimated ratios are shown next to the corresponding fit.

## The PSII_free_ contribution.

Alternatively, we can write Eq. as:

|  |  | (S23) |
| --- | --- | --- |

with:

|  |  | (S24) |
| --- | --- | --- |

Eq. (S24) helps realizing one important feature: the F_M_–level (and therefore the variable fluorescence) is determined by the sum of the differences in quantum yields between the species in closed and open states. Eq. (S6) with an additional PSII_free_ component (*f_2_*):

|  |  | (S25) |
| --- | --- | --- |

|  |  | (S26) |
| --- | --- | --- |


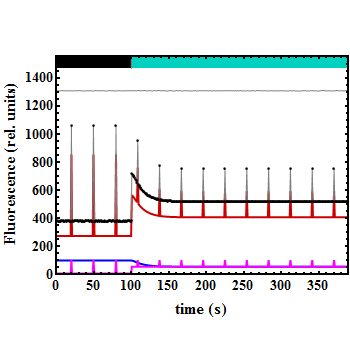


Figure S 10 Simulated data with a pulse every 30 s and noise levels of 0.01. At time t=100 s strong blue-green light is turned on. Its intensity is such that a fraction *c_0_* of the RCs gets closed and the OCP^o^→OCP^r^ conversion takes place with _I_=0.09s^-1^. The amount of OCP^r^ formed is [OCP^r^]=0.5 and it binds to PB with *k_1_*=0.30 s^-1^. Parameters have been estimated from this data in trial Q1. Key: Black dots: simulated data points; Red: PPc2-contribution; Blue: PB_free_-contribution; Magenta: PSII_free_-contribution; Gray: sum of the three contributions. Residuals are shown on top with an offset of 1300.

## Solving the linear system for three parameters

The linear system formulated in Eq. :

has a unique solution if the **A** matrix can be brought to triangular form. We study the **A** matrix by means of a simple Gaussian elimination calculation to determine under which conditions the system has a solution. Elimination of the off-diagonal elements of the first column yields:

In as second step, we would need to eliminate the third–row element of the second column to get a triangular matrix. Alternatively, we could first exchange columns 2 and 3 and go on with the elimination step by multiplying a factor that cancels out the diagonal element of the second column. The first choice yields:

Notice, however, that either way we choose to proceed, a multiplying factor is needed in which the denominator is the difference in fluorescence quantum yields of the closed and open states of either PPc2 or PSII_free_. Hence, this simple calculation shows that, unless there is neat contrast between open and closed states:

|  |  | (S27) |
| --- | --- | --- |

|  |  | (S28) |
| --- | --- | --- |

the **A** matrix cannot be brought to a triangular form and the system cannot be solved. This result was already qualitatively contained in Eq.(S24).

## The PSI contribution.

|  |  | (S29) |
| --- | --- | --- |

|  |  | (S30) |
| --- | --- | --- |

|  |  | (S31) |
| --- | --- | --- |

|  |  | (S32) |
| --- | --- | --- |


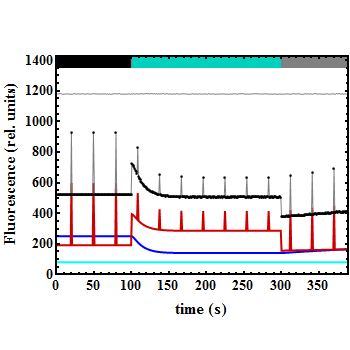


Figure S 11 Same caption as in . A PSI_free_-contribution has been added instead of PSII_free_. The relative contributions used for simulation were: *γ*=0.05, *β*=3, *scale*=5000. Estimated parameters: (*γ*=0.0498 +/- 0.0003), (*β*=3.01 +/- 0.02), (*scale*=5008.5 +/- 18.6). Key: Black dots: simulated data points; Red: PPc2-contribution; Blue: PB_free_-contribution; Cyan: PSI_free_-contribution; Gray: sum of the three contributions. Residuals are shown on top with an offset of 1300.

Note that in the simulation of Figure S 11 all NPQ parameters had to be kept fixed. When freeing those parameters, it was found that β correlates strongly with the *scale* and NPQparameters and cannot be accurately estimated due to numerical unidentifiability.

## List of parameters

γ – concentration of functionally uncoupled PB

*c_2_* – concentration of PB–PSII–complexes

*f_1_* – concentration of free PSI

*f_2_* – concentration of free PSII

*β* – proportionality factor between the number of PSI–trimers and PSII–dimers

*c_0_* – fraction of closed photosystems when turning the strong blue–green light on

*k_!_* – OCP to PB binding rate

*k_2_* – FRP detaching rate of OCP

*k_3_* – OCP^r^ deactivation rate

*_I_* – OCP^o^ to OCP^r^ activation rate

OCP^o^_0_ – initial concentration of OCP^o^

*scale* – scaling factor matching the actual fluorescence scale of the fluorometer

|  | trial Q1 | | | trial Q2 | | | trial Q3 | | | trial Q4 | | | trial Q+R | | |
| --- | --- | --- | --- | --- | --- | --- | --- | --- | --- | --- | --- | --- | --- | --- | --- |
| parameter | estimate | st. error | t-val | estimate | st. error | t-val | estimate | st. error | t-val | estimate | st. error | t-val | estimate | st. error | t-val |
| γ | 0.0509 | 0.0011 | 92.5 | 0.0496 | 0.0006 | 88.9 | 0.0505 | 0.0010 | 48.6 | 0.0505 | 0.0010 | 48.6 | 0.0504 | 0.0004 | 119.9 |
| *f_2_* | 0.1042 | 0.0041 | 49.5 | 0.0987 | 0.0021 | 47.8 | 0.1032 | 0.0049 | 21.2 | 0.1028 | 0.0049 | 21.2 | 0.1024 | 0.0015 | 68.8 |
| *c_0_* | 0.4998 | 0.0008 | 1370.3 | 0.4997 | 0.0004 | 1351.9 | 0.5003 | 0.0007 | 687.5 | 0.5002 | 0.0007 | 682.8 | 0.4999 | 0.0005 | 1059.7 |
| *scale* | 1997.67 | 4.39 | 948.6 | 2001.79 | 2.21 | 904.6 | 1999.83 | 2.94 | 680.7 | 1999.64 | 2.94 | 680.4 | 1999.53 | 1.81 | 1102.4 |
| *k_1_* | 0.3 | - | - | 0.3002 | 0.0029 | 103.2 | 0.3043 | 0.0051 | 60.0 | 0.287 | 0.014 | 20.5 | 0.2990 | 0.0033 | 90.1 |
| *k_2_* | 0.003 | - | - | 0.003 | - | - | 0.0025 | 0.0005 | 4.8 | 0.0067 | 0.0034 | 2.0 | 0.00306 | 0.00004 | 83.2 |
| OCP^o^_0_ | 0.5 | - | - | 0.5 | - | - | 0.5 | - | - | 0.514 | 0.012 | 43.5 | 0.5012 | 0.0007 | 664.7 |

Table S 2 Parameter estimation of NPQ parameters in several trials: from trial Q1-4 the kinetic parameters have been freed stepwise. The t-values, in particular that of *k_2_*, gradually drop. Adding a region of fluorescence recovery (trial Q+R) allows all parameters to be estimated with acceptable t-statistics. The Q+R trials were performed for the case of PSII_free_ being added and the amount of parameters being increased gradually. t-val: ratio of estimate and its standard error.

|  | WT  rmse = 30.9 | | | ΔApcD  rmse = 31.3 | | | ΔApcF  rmse = 27.2 | | | ΔApcDF  rmse = 27.2 | | |
| --- | --- | --- | --- | --- | --- | --- | --- | --- | --- | --- | --- | --- |
| parameter | estimate | st. error | t-val | estimate | st. error | t-val | estimate | st. error | t-val | estimate | st. error | t-val |
| γ | 0.034 | 0.001 | 28.0 | 0.059 | 0.002 | 36.0 | 0.072 | 0.002 | 46.1 | 0.131 | 0.002 | 56.8 |
| *β *(fixed) | 3.6 |  |  | 3.6 |  |  | 3.6 |  |  | 3.6 |  |  |
| *c_0_* | 0.606 | 0.011 | 54.8 | 0.564 | 0.011 | 53.1 | 0.571 | 0.011 | 50.7 | 0.577 | 0.013 | 46.0 |
| *scale* | 13899.6 | 181.2 | 76.7 | 14314.8 | 193.4 | 74.0 | 14838.5 | 177.1 | 83.8 | 13942.9 | 169.0 | 82.5 |
| *k_1_* | 0.121 | 0.010 | 11.7 | 0.243 | 0.018 | 13.7 | 0.302 | 0.031 | 9.7 | 0.451 | 0.049 | 9.3 |
| *k_2_* | 0.0096 | 0.0004 | 21.7 | 0.0068 | 0.0002 | 29.7 | 0.019 | 0.001 | 18.7 | 0.018 | 0.001 | 24.2 |
| OCP^o^_0_ | 0.61 | 0.01 | 48.9 | 0.64 | 0.007 | 88.9 | 0.53 | 0.01 | 61.2 | 0.46 | 0.01 | 91.8 |

Table S 3 Parameter estimation of experimental data. PAM was performed on whole cells of WT *Synechocystis* and mutants thereof. Estimates with their standard error and associated *t*-values. Rmse: root mean square error of the fit, t-val: ratio of estimate and its standard error.
